# New Appliances and Things Medical

**Published:** 1897-01-16

**Authors:** 


### NEW APPLIANCES AND THINGS MEDICAL.

[We shall be glad to receive, at our Office, 28 & 29, Southampton Street, Strand, London, W.C., from the manufacturers, specimens of all new preparations and appliances which may be brought out from time to time.]

### EXTRACT OF MALT, AND MALT AND MILK.

(Evans, Gadd, and Co., Exeter.)

The Extract of Malt received from the above firm has only just been put on the market. It is confidently recommended as being a carefully prepared extract, and the manufacturers guarantee its diastatic and keeping properties. Our analyst reports that the sample had a pale yellow-brown colour with a distinct malty taste, but appeared to be slightly burnt. The analysis showed the following results:—

| | |
|---|---|
| Water ... ... ... ... ... | 14·6 |
| Maltose ... ... ... ... | 61·58 |
| Mineral matter ... ... ... | 1·11 |

The above amount of maltose and ash is similar to that of other malt extracts in the market. The percentage of water represents the loss of water on drying for three hours at 212 deg. F. The diastatic power of a malt extract should be such that it digests its own weight of arrowroot starch in ten to fifteen minutes, or less at 40-42 deg. C. As this sample fulfilled the above test, the starch being entirely converted under six minutes, the diastatic value is very good. With soluble starch in the Lintner process similar satisfactory results were obtained.

The Malt and Milk of the same firm gave the following results:—

| | |
|---|---|
| Water ... ... ... ... ... | 11·2 |
| Sugar as Maltose ... ... ... | 62·67 |
| Ash ... ... ... ... ... ... | 1·32 |

The diastatic power of this sample was also under six minutes, and we understand that it contains 25 per cent. of condensed milk.

